# Optimization of novel multigrain pasta and evaluation of physicochemical properties: using D‐optimal mixture design

**DOI:** 10.1002/fsn3.2514

**Published:** 2021-08-05

**Authors:** Leila Kamali Rousta, Amir Pouya Ghandehari Yazdi, Sepideh Khorasani, Mohammad Tavakoli, Zahra Ahmadi, Mahdi Amini

**Affiliations:** ^1^ Department of Food Research and Development Zar Research and Industrial Development Group Alborz Iran; ^2^ Department of Food Science and Technology Faculty of Agriculture, Research and Technology Institute of Plant Production (RTIPP) Shahid Bahonar University of Kerman Kerman Iran; ^3^ Department of Food Science and Technology Ferdowsi University of Mashhad Mashhad Iran

**Keywords:** D‐optimal, enrichment, mixture design, multigrain, pasta

## Abstract

D‐optimal mixture design looked to be a priceless tool for optimizing the influences of semolina flour (SF), defatted soy flour (DSF), whole quinoa flour (WQF), whole rye flour (WRF), whole oat flour (WOF), whole barley flour (WBF), and rice flour (RF) on the quality attributes of multigrain pasta (MP). Multigrain flours were considered as the independent variables evaluated with respect to three response variables containing hardness and the amount of protein and fiber. Quadratic, linear, and linear models were chosen to explain the hardness and the amount of protein and fiber of the MPs, respectively. In optimal formulation of MP, that is, SF (57.34%,), DSF (14%), WQF (11%), WRF (7.54%), WOF (5.61%), WBF (2.51%), and RF (2%), the content of fiber and protein enhanced more than 4.12 and 1.34 times compared with SP, respectively. Therefore, according to the European Union law, it can be claimed that this pasta is a source of fiber. As the amount of protein and fiber increased, the hardness and optimal cooking time decreased, while the cooking loss increased. After cooking, MP was murkier and less yellow in color. The 2, 2‐ diphenyl‐ 1‐ picrylhydrazyl (DPPH) inhibition activity of the MP was about 2.5 times higher than the SP. Analysis of the antioxidant properties of the samples after cooking showed that the DPPH inhibition activity of the SP and MP reduced. The results indicated that the overall acceptability of MP was higher than SP. Based on our findings, these multigrain flours are probable to be applied as nutritious complements in the pasta industry to improve the functional characteristics.

## Graphical abstract

The fiber and protein content of multigrain pasta was higher than regular pasta. From a sensory point of view, multigrain pasta was better than semolina pasta although it was dark in color.

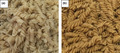

## 1 INTRODUCTION

Pasta is among one of the most favored foods being consumed globally because of its affordable price, comfortable cooking, low glycemic index, and desirable taste. Pasta is usually made from semolina, which is rich in calories but poor in dietary fibers, minerals, vitamins, and essential amino acids (Ghandehari Yazdi et al., 2020). Researches have indicated that the matrix of pasta has the capability to maintain the stability of the nutrients and can be an appropriate carrier to enrich the food components (Kamble et al., 2019). Due to the health benefits of multigrain foods such as slow digestion, cholesterol‐lowering effect, and antioxidant, anti‐carcinogenic and anti‐inflammatory activities, their consumption has increased among consumers (Saleh et al., 2013). Within the previous decade, some fascinating research has been conducted to increase the nutritional potential of pasta by mixing the flour of different cereals such as quinoa and faba bean flour (Rosa‐Sibakov et al., 2016), fermented quinoa flour (Lorusso et al., 2017), plant proteins out of mushroom powder, Bengal gram flour and defatted soy flour (DSF) (Kaur et al., 2013), soybean and sweet potato (Marengo et al., 2018), and sorghum flour and finger millet flour (Kamble, Singh, Rani, Kaur, et al., 2019). In this study, the combination of quinoa (*Chenopodium quinoa*), oat (*Avena sativa L*), barley (*Hordeum vulgare*), rye (*Secale cereale*), rice (*Oryza sativa*), and Defatted soy (*Glycine max*) flours were used to enrich the pasta. Each of these compounds has its own functional properties. For example, oat and barley flours are great sources of dietary fiber, especially beta‐glucan that saves people from diabetes, cardiovascular disease, blood cholesterol, and excessive weight gain. Oat contains more protein than other grains do. Additionally, it is an excellent source of vitamins, minerals, and natural antioxidants (Rasane et al., 2015). Barley is a rich source of protein, unsaturated fatty acids, vitamins (such as thiamine and niacin), antioxidants (such as lignin's phenolic compounds), and minerals (Panfili et al., 2003). Quinoa is considered to be a thorough protein source, which means it can provide the whole essential amino acids. It has better protein than most grains. The quality of quinoa protein is comparable to milk casein (Comai et al., 2011). Rye contains vitamins, fibers, minerals, and essential amino acids. Soy is one of the best sources of plant protein (36%–56% of dry weight). The nutritional value of soy protein is considerable and is somewhat comparable to animal proteins. Rice also has been proved to be a good source of minerals (manganese, selenium, magnesium, and copper) and vitamins (thiamin and niacin) (Runge et al., 2019).

Fortification with these components is an efficient method to increase the nutritional attributes of pasta; however, it presents a challenge because of their effects on the texture, cooking, and sensory properties of pasta (Kamali Rousta et al., 2020). Meanwhile, using the Mixture design methodology might be considered a useful tool to investigate the role of each component in processed foods and accents the significance of component interactions (Arteaga et al., 1993).

The aims of this study were (a) to investigate the possibility of producing new functional pasta using a combination of different cereal flour, (b) to use the D‐optimal mixture design to obtain optimal formulation based on nutritional and rheological properties of multigrain pasta (MP), and (c) to study the physicochemical, nutritional, and sensory attributes of optimal formulation of MP. To the best of our knowledge, our experiment is the pioneer study in the literature concentrated on the production of MP by the mixture of semolina flour (SF), DSF, whole quinoa flour (WQF), whole rye flour (WRF), whole oat flour (WOF), whole barley flour (WBF), and rice flour (RF).

## 2 MATERIALS AND METHODS

### 2.1 Chemical and raw materials

Semolina was obtained from Zar Semolina Co. (Alborz, Iran), rye, rice, and soy flour were purchased from local market (Tehran, Iran). Oat and quinoa flour (Organic Bolivian quinoa) were purchased from Iranian Health‐Based Biotechnology Co. (Tehran, Iran). Flours were sieved to pass through an 840 µm mesh screen. All the used chemicals were provided from Sigma Aldrich (Milan, Italy).

### 2.2 Experimental design and statistical analysis

The Design‐Expert software version 9.0.4 (Stat‐Ease Inc., Minneapolis, MN, USA) was implemented to design the multigrain formulation. In this research D‐optimal design was applied with seven ingredients: semolina flour (SF), defatted soy flour (DSF), whole quinoa flour (WQF), whole rye flour (WRF), whole oat flour (WOF), whole barley flour (WBF), and rice flour (RF). Table 1 displays the formulations calculated by the experimental design. The range of each ingredient was selected based on initial tests and the ingredients ranges were as follows: SF: 50%–80%, DSF: 4%–14%, WQF: 4%–12%, WRF: 2%–8%, WOF: 2%–8%, WBF: 2%–8%, and RF: 2%–8%. 38 formulations were designed by Design‐Expert software (Table 1). SF, DSF, WQF, WRF, WOF, WBF, and RF on properties of product were studied and optimum formulation was chosen. After optimizing the formulation of MP based on protein, fiber, and texture, its cooking features, color, antioxidant activity, and sensory were compared with the control pasta (SP).

**TABLE 1:** Composition of multigrain‐formulated blend

RF	WBF	WOF	WRF	WQF	DSF	SF	Run
8	2	2	2	12	4	70	1
8	8	8	8	4	14	50	2
2	2	2	2	12	14	66	3
6	8	8	2	12	14	50	4
2	2	2	8	4	9	67	5
2	2	2	8	12	4	70	6
2	8	8	8	12	14	50	7
6	2	2	8	12	14	50	8
5.082	5.082	5.082	5.082	8.472	9.506	61.694	9
8	2	2	8	4	4	72	10
2	2	2	2	8	4	80	11
2	8	8	8	4	4	72	12
2	8	8	2	4	14	68	13
2	4	4	2	4	6	80	14
8	2	2	2	12	4	64	15
2	8	8	2	12	4	70	16
2	2	2	8	12	14	57	17
8	5	5	2	12	14	57	18
2	2	2	8	12	4	70	19
2	8	8	2	4	4	72	20
2	2	2	2	4	14	68	21
2	2	2	8	4	14	68	22
5.082	5.082	5.082	5.082	8.472	9.506	61.694	23
2	2	2	8	4	9	67	24
2	8	8	6	12	14	50	25
8	8	8	8	12	4	52	26
8	8	8	8	4	4	60	27
2	8	8	2	12	4	70	28
2	5	5	8	4	14	59	29
8	2	2	2	4	4	72	30
8	2	2	2	4	14	68	31
8	8	8	5	4	14	59	32
8	2	2	8	12	9	59	33
2	2	2	2	8	4	80	34
2	2	2	2	12	4	70	35
6	8	8	8	12	14	50	36
8	8	8	2	4	4	72	37
2	2	2	4	4	4	80	38

Abbreviations: DSF, defatted soy flour; RF, rice flour; SF, semolina flour; WBF, whole barley flour; WOF, whole oat flour; WQF, whole quinoa flour; WRF, whole rye flour.

### 2.3 Pasta preparation

The pasta was prepared according to the formula of Jalgaonkar et al., (2018). For this purpose, 600 ml of water was added to semolina (2 kg) with continuous mixing (10 min) in the chamber of pasta extruder (Anselmo, Bene Vagienna, Italy). The blend was then extruded at 25℃. Finally, the extruded pasta was dehydrated in a cabinet dryer (Anselmo, Bene Vagienna, Italy) at 75 ± 2℃ for 5 h to attain the moisture content of 8%–10%. For the preparation of MP, semolina was substituted by other cereal flours (Table 1).

### 2.4 Chemical analyses

To determine moisture content, crude protein, fat, fiber, and ash of flours and pasta products (MP and SP), the AACC methods (American Association of Cereal Chemists, 2000) were used. Results were reported based on dry weight (g/100 g).

### 2.5 Cooking characteristics of pasta

The optimal cooking time (OCT) and cooking loss were determined based on the procedure described by Tudorica et al., (2002).

### 2.6 Color

The color values of products were evaluated implementing a Hunter Color flex colorimeter (Hunter Lab, USA) by determining L* (black (0) to white (100)), a* (+a = red, ‐a = green), and b* (+b= yellow, ‐b =blue) values (Ghandehari Yazdi et al., 2017).

### 2.7 Textural analysis

Texture profile Analyzer TA.XT plus (Stable Micro System, Reading, UK) equipped with a steel cylindrical probe (p/75 mm) was used to determine the textural properties of cooked pasta (in OCT). The textural parameters were adjusted according to the method described by Kamali Rousta et al. (2020). During the first compression, hardness was described as the highest compression force (Ghandehari Yazdi et al., 2017; Rosa‐Sibakov et al., 2016).

### 2.8 Antioxidant activity assessments

Free radical scavenging activity for samples was conducted using 2, 2‐ diphenyl‐ 1‐ picrylhydrazyl (DPPH)^•^ assay according to the protocol of Gull et al., (2016). The percentage of inhibition was calculated by the equation (1):

(1) $$\text{Inhibition}\%=\left(A_{\text{517 Control}}‐A_{\text{517 Sample}}\right)/A_{\text{517 Control}}\times 100,$$

where (*A*_Control_) is the absorbance of the control reaction and (*A*_Sample_) is the absorbance of the test samples. The absorbance was observed at 517 nm.

### 2.9 Sensory evaluation

Sensory evaluation of the cooked samples was carried out by thirty trained panelists (15 males and 15 females with ages ranging from 20 to 35 years) that were selected from Zar Co. employees. The samples (50 g of MP and SP) were cooked at the optimum time in 250 ml boiling water. Panelists were asked to present their liking scores from 1 to 9 (1: extremely undesirable, to 9: extremely desirable) on texture, flavor, color, and overall quality (Biró et al., 2019).

### 2.10 Statistical and data analysis

Linear and quadratic models were investigated (eq. (2) and (3)) and all the responses (*Y*) with the independent variables were fitted by these two models.

(2) Y=b1X1+b2X2+b3X3+b4X4+b5X5+b6X6+b7X7,

(3) $$\begin{array}{cc} Y= & \text{b1X1}+\text{b2X2}+\text{b3X3}+\text{b4X4}+\text{b5X5}+\text{b6X6}+\text{b7X7}+\text{b8X1X2}+\text{b9X1X3}+\text{b1}0\text{X1X4}+\text{b11X1X5}\\ & +\text{b12X1X6}+\text{b13X1X7}+\text{b14X2X3}+\text{b15X2X4}+\text{b16X2X5}+\text{b17X2X6}+\text{b18X2X7}+\text{b19X3X4}+\text{b2}0\text{X3X5}\\ & +\text{b21X3X6}+\text{b22X3X7}+\text{b23X4X5}+\text{b24X4X6}+\text{b25X4X6}+\text{b26X5X6}+\text{b27X5X7}+\text{b28X6X7,} \end{array}$$

where X1is SF, X2 is DSF, X3 is WQF, X4 is WRF, X5 is WOF, X6 is WBF, X7 is RF, and b are the regression coefficients calculated from the experimental data by multiple regressions.

All experiments were done in triplicate. Fisher's least significant differences test was applied to estimate the significant differences at 95% confidence level. Statistical analysis was done by SAS 9 (Institute Inc, Carolina, USA) software.

## 3 RESULT AND DISCUSSION

### 3.1 Chemical and nutritional composition of raw materials

Table 2 presents the chemical composition of the raw materials of Multigrain. Based on the results, the highest protein value was observed in DSF. Protein type and amount play a critical role in the texture, cooking properties, and nutritional value of pasta. Fiber improves the nutritional value of pasta, and its maximum amount was found in WOF, WRF, and WQF, respectively.

**TABLE 2:** Chemical composition of the raw material of multigrain

Chemical Analysis (%)	SF	DSF	WQF	WRF	WOF	WBF	RF
Moisture	13.80 ± 0.44	7.12 ± 0.34	13.26 ± 0.15	13.9 ± 0.18	11.55 ± 0.55	8.01 ± 0.03	8.94 ± 0.06
Protein	12.05 ± 0.61	45.62 ± 0.60	14.12 ± 0.11	12.01 ± 0.11	12.82 ± 0.27	9.25 ± 0.02	7.99 ± 0.11
Fat	1.10 ± 0.13	1.67 ± 0.01	5.88 ± 0.09	1.09 ± 0.1	5.77 ± 0.23	1.94 ± 0.05	1.54 ± 0.11
Total ash	0.74 ± 0.05	3.86 ± 0.11	2.38 ± 0.13	0.76 ± 0.2	2.14 ± 0.13	2.32 ± 0.01	0.52 ± 0.07
Crude Fiber	0.95 ± 0.28	3.20 ± 0.09	5.10 ± 0.09	6.96 ± 0.03	19.33 ± 0.76	4.30 ± 0.06	4.01 ± 0.01

Abbreviations: DSF, defatted soy flour; RF, rice flour; SF, semolina flour; WBF, whole barley flour; WOF, whole oat flour; WQF, whole quinoa flour; WRF, whole rye flour.

### 3.2 Fitting for the best model

**FIGURE 1:**
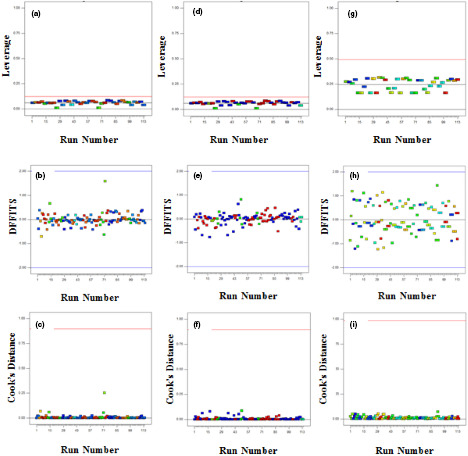
Leverages, difference in fits (DFFITS), and Cook's distance for protein (a, b, and c), fiber (d, e, and f), and hardness (g, h, and i) models

Protein and fiber content and hardness of the samples were explained by linear, linear and quadratic models, respectively. Depending on low standard deviation, minimum predicted sum of squares, and high *R*‐squared, the best model was chosen (Kamali Rousta et al., 2020). *p*‐values and lack of fit *p*‐values of the optimal model were <.05 and >.05, respectively. The sufficient precision values of models were more than 4 and it can be concluded that the models can be applied to monitor the design space (Diedericks & Jideani, 2015). Figure 1(a–i), indicates the difference in fits (DFFITS), Leverages, and Cook's distance for hardness, fiber, and protein. As can be seen, all of the leverage values are lower than .50, so there are no outliers or unanticipated errors in the model. Also, the reliability of the model was confirmed by Cook's distance and DFFITS plots because the values are within the specified range (Jalali‐Heravi et al., 2009).

### 3.3 Protein

According to Table 3, SF, DSF, WQF, WRF, WOF, WBF, and RF with their positive coefficient had significantly improved the protein content of pasta. The greatest effect on protein content was related to DSF. As demonstrated in Table 4, the protein content ranged between 12.91% and 16.85%. The maximum content of protein was observed in the formulation consisting of 66% SF, 14% DSF, 12% WQF, 2% WOF, 2% WRF, 2% WBF, and 2% RF flours (run 3, protein content: 16.85). According to the findings, the mixture of these ingredients improved the protein content of MP by 35% compared to the SP. Enhancing the protein content of pasta using the similar ingredients has been previously reported by others (Kaur et al., 2011; Lorusso et al., 2017; Sereewat et al., 2015). For example, Shogren et al., (2006) reported that enrichment of pasta with soy (at the level of 50%) increased protein content by 54% compared to the control sample (without soy flour). In addition, Lorusso et al., (2017) indicated that the addition of quinoa flour (at the level of 20%) to pasta enhanced the protein content by 20% in comparison with the control sample (without quinoa flour). Recently, Rani et al., (2020) declared that the amount of protein in multigrain noodles (19.10 ± 0.63) formulated with the mixture of sorghum (24.60%), soy flour (13.20%), and refined wheat (62.20%) was higher than refined wheat noodles (14.82 ± 0.95). However, increasing the content of protein could be a consequence of the higher content of protein in DSF and WQF than the SF. Kamali Rousta et al., (2020) suggested that a complete amino acid profile could be obtained by combining legumes with cereals flours because the content of sulfur‐containing amino acids in legume proteins is low, while they are rich in proteins and essential amino acids such as lysine.

**TABLE 3:** Regression coefficients and correlation for the adjusted model to experimental data in D‐optimal mixtures design for protein, fiber, and hardness of pasta

Variable	Protein	Fiber	Hardness
A	.120^a^	.010^a^	.190^a^
B	.460^a^	.030^a^	1.760^a^
C	.140^a^	.071^a^	−.290^a^
D	.110^a^	.152^a^	4.100^a^
E	.130^a^	.201^a^	−2.400^a^
F	.094^a^	.046^a^	−2.250^a^
G	.078^a^	.039^a^	1.370^a^
AB	‐	‐	−.024^a^
AC	‐	‐	.001^c^
AD	‐	‐	−.046^a^
AE	‐	‐	.026^a^
AF	‐	‐	.025^a^
AG	‐	‐	−.016^a^
BC	‐	‐	−.017^a^
BD	‐	‐	−.071^a^
BE	‐	‐	.005^a^
BF	‐	‐	.002^b^
BG	‐	‐	−.035^a^
CD	‐	‐	−.035^a^
CE	‐	‐	.031^a^
CF	‐	‐	.032^a^
CG	‐	‐	−.013^a^
DE	‐	‐	−.021^a^
DF	‐	‐	−.034^a^
DG	‐	‐	−.062^a^
EF	‐	‐	.050^a^
EG	‐	‐	.005^b^
FG	‐	‐	.011^a^
*R* ^2^_perd_	.990	.990	.990
LOF^d^	.520	.760	1.810
AP^e^	204.770	230.138	798.920

Note

Semolina flour (A), defatted soy flour (B), whole quinoa flour (C), whole rye flour (D), whole oat flour (E), whole barley flour (F), Rice flour (G).

^a^ Significant at .0001 levels.

^b^ Significant at .01 levels.

^c^ Not Significant at .05 levels.

^d^ Lack of fit.

^e^ Adequate precision.

**TABLE 4:** Protein, fiber, and texture as a response to various runs of multigrain

Run	Protein%	Fiber%	Hardness (*N*)
1	13.239 ± 0.114	2.686 ± 0.063	8.820 ± 0.010
2	16.201 ± 0.135	4.616 ± 0.043	3.850 ± 0.010
3	16.850 ± 0.051	2.721 ± 0.086	7.230 ± 0.010
4	16.551 ± 0.084	4.208 ± 0.027	4.393 ± 0.006
5	15.053 ± 0.129	4.097 ± 0.034	7.830 ± 0.010
6	13.385 ± 0.041	3.333 ± 0.085	9.390 ± 0.010
7	16.644 ± 0.059	4.567 ± 0.005	4.473 ± 0.006
8	16.623 ± 0.035	4.810 ± 0.041	4.233 ± 0.006
9	15.040 ± 0.059	3.663 ± 0.117	6.450 ± 0.010
10	12.967 ± 0.065	3.047 ± 0.033	10.020 ± 0.010
11	13.430 ± 0.047	2.281 ± 0.037	11.510 ± 0.010
12	13.045 ± 0.038	3.046 ± 0.053	9.880 ± 0.010
13	16.497 ± 0.019	2.477 ± 0.011	8.323 ± 0.006
14	13.948 ± 0.069	2.103 ± 0.079	11.543 ± 0.006
15	13.304 ± 0.008	3.840 ± 0.009	7.430 ± 0.010
16	13.293 ± 0.059	2.780 ± 0.060	9.343 ± 0.006
17	16.768 ± 0.079	4.207 ± 0.106	5.993 ± 0.006
18	16.493 ± 0.027	3.011 ± 0.034	6.023 ± 0.006
19	13.423 ± 0.037	3.376 ± 0.010	9.393 ± 0.006
20	13.191 ± 0.053	3.342 ± 0.018	10.193 ± 0.006
21	16.720 ± 0.031	3.355 ± 0.026	8.153 ± 0.006
22	16.606 ± 0.023	3.119 ± 0.007	8.047 ± 0.006
23	15.052 ± 0.187	3.655 ± 0.108	6.453 ± 0.006
24	15.059 ± 0.324	4.132 ± 0.019	7.823 ± 0.006
25	16.743 ± 0.066	4.667 ± 0.027	4.003 ± 0.006
26	13.056 ± 0.069	4.944 ± 0.045	5.253 ± 0.006
27	12.910 ± 0.056	4.415 ± 0.017	6.813 ± 0.006
28	13.392 ± 0.061	2.730 ± 0.015	9.343 ± 0.006
29	16.602 ± 0.056	4.373 ± 0.052	6.163 ± 0.006
30	13.147 ± 0.017	3.301 ± 0.056	9.737 ± 0.006
31	16.444 ± 0.004	2.450 ± 0.005	8.217 ± 0.012
32	16.257 ± 0.055	3.103 ± 0.021	5.833 ± 0.115
33	14.816 ± 0.027	3.680 ± 0.007	6.033 ± 0.006
34	13.438 ± 0.043	2.284 ± 0.024	11.513 ± 0.006
35	13.535 ± 0.013	3.638 ± 0.016	8.983 ± 0.006
36	16.425 ± 0.049	3.951 ± 0.059	4.247 ± 0.006
37	12.983 ± 0.061	2.418 ± 0.069	10.213 ± 0.006
38	13.299 ± 0.018	2.674 ± 0.025	11.747 ± 0.006
C	12.480 ± 0.510	1.010 ± 0.021	12.641 ± 0.050

Note

Data are the means ±standard of three replicates.

### 3.4 Fiber

As the fiber content investigation showed, WOF presented an influential effect on the pasta fiber content, DSF, WQF, WRF, WBF, and RF also increased it. According to Table 4, fiber contents varied between 2.10% and 4.94%. MP displayed a significantly (*p* < .0001) greater fiber content in comparison with the control. The highest amount of fiber was observed with the combination of 52% SF, 4% DSF, 12% WQF, 8% WOF, 8% WRF, 8% WBF, and 8% RF flours (run 26). Combination of these compounds increased the amount of fiber by 4.89‐fold compared to the control sample. Increasing the fiber content of MP formulated with different compounds compared to semolina pasta (control) has been reported by other authors (Kamble, Singh, Rani, Kaur, et al., 2019; Rani et al., 2018). Kamble, Singh, Rani, Kaur, et al., (2019) reported that addition of finger millet (13.04%), sorghum (31.96%), and gluten (3.40%) to pasta enhanced the fiber content about 60%. Established MP may be acclaimed as a fiber‐rich product, which provides more different health benefits regarding the consumption of dietary fiber‐rich products (Banerjee et al., 2014) such as optimal digestive health, satiety promotion, postprandial insulin response modulation, cholesterol and lipid absorption decreases, endogenous cholesterol alteration to bile acids improvement, and decrease of possibility and severity of gastrointestinal infection and inflammation (Montemurro et al., 2019). According to regulation (EC) No. 1924/2006 of the European Parliament and of the Council of 20 December 2006, high‐fiber food such as high‐fiber pasta can be claimed as a source of fiber and high‐fiber content when the content of fiber in 100 g of product is at least 3 and 6 g, respectively (Herrera et al., 2010).

### 3.5 Hardness

Texture is among one of the most critical properties of pasta. Table 4 displays the hardness of the pasta varied between 3.85 (run2) and 11.74 N (run 38). The results in Table 3 show that WRF, DSF, and RF with their positive coefficients had significantly (*p* < .0001) increased hardness, while WBF, WOF, and WQF with their negative coefficients reduced this factor in the pasta. However, WRF, DSF, and RF had the highest effect on the texture of MP, respectively. As shown in Table 3, the interaction of SF/WOF, SF/WBF, DSF/WOF, WQF/WOF, WQF/WBF, WOF/WBF, and WBF/RF showed positive coefficients, which indicated the hardness increase (*p* < .0001). The hardness of run 38 was similar to the control, while the hardness of run 2 was 3.28 times lower than the control sample. In general, hardness is reduced by replacing semolina with other compounds. Contrary to these results, many researchers indicated that by increasing the protein content, the value of hardness of this product increased (Kamali Rousta et al., 2020; Teterycz et al., 2020). Mudgil et al. (2016) suggested that the hardness of pasta is related to the gluten network within dough development and extrusion process. The obtained results could be a consequence of gluten network dilution simultaneously with the reduction of accessibility to water to expand the gluten network (De Pilli et al., 2013). La Gatta et al., (2017) asserted that the rivalry among the fiber, protein, and starch is effective on the structure of gluten network. In addition, foreign proteins that interfere with the formation of the gluten‐starch complexes may reduce the hardness. Also, Tudorica et al., (2002) declared that by augmenting the amount of fiber, the hardness of pasta decreased. They revealed that the decrease in hardness of the pasta is related to the role of fiber in interrupting the starch‐protein complex (Tudorica et al., 2002). These results are in agreement with the reports of Edwards et al., (1995). The results of this study showed that the hardness depends on the level, kind, and interaction of the flours incorporated with the product.

### 3.6 Optimization

Optimization was done by maximizing the amount of protein and fiber, at the same time, to keep hardness in normal range of 8–12.80 N (due to the tolerable texture). The optimal content of SF, DSF, WQF, WRF, WOF, WBF, and RF were 57.34%, 14%, 11%, 7.54%, 5.61%, 2.51%, and 2%, respectively. MP in this condition had protein content of 16.77%, a fiber content of 4.17, and a hardness of 8.2 N. The amount of protein and fiber in the optimal sample were 1.34 and 4.12 times higher than the control sample. The desirability score of chosen mixture was .92. The function of desirability transforms an estimated response to a scale‐free value (Harrington, 1965). Sarteshnizi et al., (2015) reported that the desirability value of more than .8 is desirable showing that the quality of samples is admitted.

### 3.7 Cooking properties of semolina and multigrain pasta

Cooking attributes of pasta considerably influence the modality of pasta (Kamali Rousta et al., 2020). According to Table 5, the OCT for the SP was more than the MP. These results supported the texture test data that showed the hardness of SP was higher than MP, and therefore, more time is needed to cook the SP. Similar results have been reported by Kaur et al., (2017) who evaluated the replacing of wheat flour with multigrain flour (soybean, mung bean, millets, barley, maize, oats, and flaxseeds) to produce MP. On the contrary, a positive linear correlation between the amount of protein and OCT was reported (Kamali Rousta et al., 2020). Park and Baik (2009) suggested that the OCT depends on various parameters such as amount of protein, quality of gluten, and strength of the flour. However, this result could be related to the interruption of the gluten network by the fiber particles, which provided a path of water penetration into the pasta that decreased cooking time.

**TABLE 5:** Physicochemical, nutritional, and sensory properties of control and optimum multigrain pasta

Samples	SP	MP
Chemical properties (%)		
Protein (%)	12.48 ± 0.50^b^	16.77 ± 0.50^a^
Fat (%)	0.98 ± 0.11^b^	1.96 ± 0.22^a^
Total ash (%)	0.74 ± 0.06^b^	1.63 ± 0.10^a^
Crude fiber (%)	1.01 ± 0.20^b^	4.17 ± 0.21^a^
Moisture (%)	9.60 ± 0.20^a^	9.72 ± 0.27^a^
Color analysis		
L*	72.18 ± 0.15^a^	64.80 ± 0.14^b^
a*	4.97 ± 0.02^b^	6.85 ± 0.11^a^
b*	32.81 ± 0.06^a^	29.00 ± 0.10^b^
Cooking properties		
OCT (min)	14.00 ± 0.50^b^	10.00 ± 0.50^a^
Cooking loss (%)		
	6.37 ± 0.20^b^	7.85 ± 0.13^a^
DPPH inhibition (%)		
Uncooked	18.63 ± 0.73^b^	47.18 ± 0.86^a^
Cooked	14.23 ± 0.03^b^	35.24 ± 0.37^a^
Sensory evaluations		
Flavor	7.66 ± 0.37^b^	8.73 ± 0.18^a^
Texture	7.58 ± 0.12^b^	8.42 ± 0.33^a^
Color	8.76 ± 0.19^a^	7.78 ± 0.62^b^
Overall liking	7.84 ± 0.12^b^	8.82 ± 0.36^a^

Note

Data are the means ±standard of three replicates. Values with different lowercase letters (a and b) are significantly different in the rows (LSD, *p* < .05).

Abbreviation: MP: multigrain pasta, OCT: optimal cooking time, SP: semolina pasta.

Cooking loss is used to evaluate the performance of pasta during cooking, and its value should not be more than 8% (Teterycz et al., 2020). According to Table 5, the cooking loss of the MP was about 23% more than SP. Similar results were observed by other researchers (Kamble, Singh, Rani, Kaur, et al., 2019; Kaur et al., 2017). The negative correlation between cooking loss and amount of protein reported by Biernacka et al., (2018). Also, Laleg et al. (2017) suggested the cooking loss increased by reduction of the gluten. Moreover, these results may be related to increased amount of fiber in the product. In fact, increased amount of fiber in pasta may prevent the gluten matrix expansion. This could lead to an increase in the vulnerability of starch and other ingredients to being solved in boiling water during cooking (Kaur et al., 2017; Teterycz et al., 2020).

### 3.8 Antioxidant activity of semolina and multigrain pasta

According to Table 5, MP had significantly higher DPPH inhibition activity (47.18 ± 0.86%), compared with SP (18.63 ± 0.73%). A similar trend was reported by others (Kamble et al., 2021; Kamble, Singh, Rani, Kaur, et al., 2019; Rani et al., 2018; Rani et al., 2020). For instance, Kamble et al., (2021) showed that the antioxidant activity of pasta prepared with a mixture of sorghum flour, semolina, and finger millet flour was higher than control (semolina). Also, Rani et al., (2020) indicated that DPPH inhibition activity of noodles formulated with sorghum, wheat, and soy flour was higher than noodles prepared with wheat flour. These results could be attributed to the attendance of different flours in MP formulations that have higher antioxidant properties compared to semolina (Gull et al., 2016; Kamble et al., 2021; Montemurro et al., 2019).

Analysis of the antioxidant properties of the samples after cooking showed that the DPPH inhibition activity of the SP and MP reduced. However, the inhibitory power of MP (35.24 ± 0.37) was higher than SP (14.23 ± 0.03). These results are consistent with the findings of Kamble et al., (2021), who reported a significant reduction in the antioxidant activities of multigrain (mixture of Semolina, sorghum flour, finger millet flour) and control (semolina) pasta after cooking. The decreased antioxidant activity in cooked pasta compared to uncooked pasta was also reported in other studies (Gull et al., 2016). These findings could be the consequence of bioactive compounds leaching into water, as well as the thermal degradation of these compounds during cooking (Hirawan et al., 2010). The higher reduction of antioxidant properties in cooked MP compared to cooked SP may be related to the structure of MP.

### 3.9 Color analysis of semolina and multigrain pasta

**FIGURE 2:**
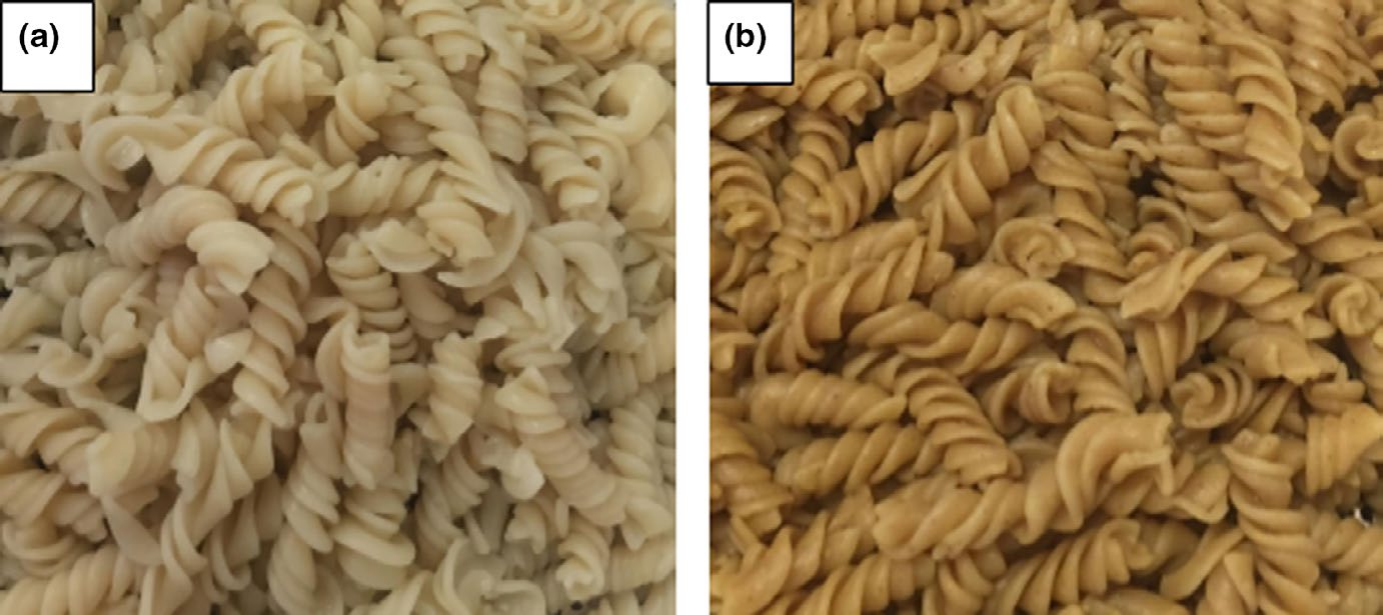
Color of semolina pasta (a) and multigrain pasta (b)

Color occupies an effective role in the appearance of the pasta and actively influences the consumer's decision to buy the product (Ghandehari Yazdi et al., 2020). The color properties in terms of a* (red (+) / green (−)), L* (black (0) / white (100)), and b* (yellow (+) / blue (−)) values of MP and SP are indicated in Table 5. According to Figure 2 and Table 5, the substitution of semolina by multigrain flours in pasta formulation resulted in significant decreases of L* and b* and a contrary trend was observed for a*. Our results are in agreement with Kamble, Singh, Rani, Kaur, et al., (2019) who investigated the substitution of the drum flour with various ingredients (sorghum flour, finger millet flour, and gluten) to produce MP. Also, a decrease in brightness and yellowness in MP compared to the wheat pasta has been reported by Kaur et al., (2017). This result could be attributed to the improved amount of fiber in pasta (Kaur et al., 2017). In addition, the decrease in L* of MP could be a consequence of the higher content of ash and color properties in the used flours (Teterycz et al., 2020).

### 3.10 Sensory properties of the final cooked semolina and multigrain pasta

The texture, color, and taste are the important features for admissibility. As can be seen from Table 5, the highest scores of overall liking, taste, and hardness were found in MP. While from a color point of view, the score of SP was higher than MP. This is probably related to the darker color of MP compared to SP. However, increasing the overall acceptability score of the MP could be due to the desired taste and texture of this sample. Despite these results, a reduction in the desirability of pasta sensory scores has been reported by the addition of similar ingredients such as soy flour (Shogren et al., 2006) and quinoa flour (Demir & Bilgiçli, 2020). Also, La Gatta et al., (2017) suggested that the fortification of pasta with high‐fiber ingredients may cause a dilution of the gluten‐protein matrix and result in an adverse effect on its sensory feature.

## 4 CONCLUSION

The formulation of MP was optimized by D‐optimal mixture design. The optimal formulation contained 57.34% SF, 14% DSF, 11% WQF, 7.54% WRF, 5.61% WOF, 2.51% WBF, and 2% RF. The amount of fiber and protein in MP was 4.12 and 1.34 times higher than the SP, respectively. By increasing the amount of protein and fiber, the hardness and OCT of MP decreased and its cooking loss increased. From a sensory point of view, the overall acceptability of MP was better than SP. MP has substantial potential as a fiber‐protein‐rich supplementary food to enhance the nutrient delivery. Due to high amount of fiber in MP, this product may be claimed as a source of fiber, which provides different health benefits associated with consumption of dietary fiber‐rich products.

## CONFLICT OF INTEREST

The authors declare that there is no conflict of interest that could be perceived as prejudicing the impartiality of the research reported.

## AUTHOR CONTRIBUTION

**Leila Kamali:** Data curation (equal); Investigation (equal); Methodology (equal); Resources (equal); Software (equal); Writing‐original draft (equal). **Amir Pouya Ghandehari Yazdi:** Conceptualization (equal); Project administration (equal); Resources (equal); Supervision (equal); Validation (equal); Visualization (equal); Writing‐review & editing (equal). **Sepideh Khorasany:** Data curation (equal); Methodology (equal); Resources (equal); Writing‐original draft (equal). **Mohammad Tavakoli:** Data curation (equal); Formal analysis (equal); Investigation (equal); Methodology (equal); Writing‐original draft (equal). **Zahra Ahmadi:** Data curation (equal); Formal analysis (equal); Investigation (equal); Methodology (equal); Software (equal); Writing‐original draft (equal). **Mehdi Amini:** Data curation (equal); Formal analysis (equal); Investigation (equal); Methodology (equal); Software (equal); Writing‐original draft (equal).
